# *In vitro* capacity and *in vivo* antioxidant potency of sedimental extract of *Tinospora cordifolia* in streptozotocin induced type 2 diabetes

**Published:** 2013

**Authors:** Ramachandran Kannadhasan, Subramaniam Venkataraman

**Affiliations:** 1 ***Research Associate, Dr.C.L.Baid Metha Foundation for Education and Research, Thoraipakkam, Chennai-600 097, Tamil nadu, India***

**Keywords:** Antioxidants, Sedimental Extract of *Tinospora Cordifolia* (SETc), Streptozotocin (STZ), Type 2 Diabetes

## Abstract

**Objective:** The role of herbs against the free radicals have been put forth recently in combating many diseases. The aim of this study was to elucidate the *in vitro* capacity and *in vivo* antioxidant properties of sedimental extract of *Tinospora cordifolia* (SETc).

**Materials and Methods: **SETc was subjected to *in vitro* chemical analysis such as 1,1-diphenyl-2-picrylhydrazyl (DPPH), nitric oxide, hydrogen peroxide, and superoxide anion radicals scavenging respectively and finally drugs reductive ability in order to elucidate the antioxidant capacity of the test drug before introducing it into the biological membrane. The resulting capacity was evaluated *in vivo* by analyzing enzymic (SOD, CAT) and non-enzymic (vitamin C & E) antioxidant levels in the homogenized samples of major organs isolated from streptozotocin induced type 2 diabetic rats after 30^th^ day of SETc (1000 mg/kg/p.o.) treatment. Finally, the histopathological evaluation was done using cut portion of the respective organs prone to free radical mediated cell destruction with STZ in order to study their micro anatomical changes.

**Results:** Chemical analysis with SETc *in vitro* for its IC_50_ proves a key evident for its total antioxidant capacity of around 2046 times, in 1000 mg/kg of fixed dose per oral for *in vivo* analysis. In contrast to the above, the lipid peroxide levels and *in vivo* enzymic and non-enzymic antioxidant levels were found to possess most significant difference (p<0.001) and moderate difference (p<0.01) with diabetic non-treated animals which was an supporting contribution for those *in vitro* parameters studied and have proved that SETc (1000 mg/kg/p.o.) was a potent drug to elevate the antioxidants levels and further healing of damaged organs as compared with that of diabetic and standard drug treated groups.

**Conclusions:** Finally, it was concluded that, the presence of antioxidant potentials in SETc was about 2046 time as an effective scavenger of free radicals *in vitro* and as a potent healer in ameliorating many signs of tissue damages *in vivo* in long term complicated diseases such as diabetes.

## Introduction

Oxygen free radicals are natural physiological products, but are also reactive species. Free radicals generated *in vivo*, damage everything found in living cells including proteins, carbohydrates, DNA, and other molecules in addition to lipids, .i.e., oxidizable substrate. Hyperglycemia alone does not cause complications resulted from chronic glucose toxicity (Hideaki et al., 1999 ▶) which is mediated and complicated through oxidative stress (Robert D. Hoeldtke et al., 2005 ▶). Hence, antioxidants are any substance that, when present at low concentrations compared to those of an oxidizable substrate, delay or prevent oxidation of that substrate.

Lipid Peroxides (LPO) and deficiency of enzymes like Superoxide Dismutase (SOD), Catalase (CAT) and non-enzymes such as vitamin C and E are important factors in the development of diabetic complications *in vivo*. Many other substances have been proposed to act as anti-oxidants *in vivo*. They include β-carotene, other carotenoids, xanthophylls, metallothioein, taurin and its precursors, creatinine, polyamines, retinol, flavonoids, and other phenolic compounds of plant origin (Dhanukar, Kulkarni, Rege, 2000 ▶).

Synthetic drug molecules with established mode of actions, rarely exhibit antioxidant activity and high incidence of toxicity in long term treatment and are well documented. Herbal molecules have the distinct advantages of built-in antioxidant activity which have an important therapeutic impact to prevent the late complications of diabetes, such as nephropathy and cardiovascular diseases (Chopra and Singh, 1994 ▶).

Indian medicinal plants of different species have been reported to have antidiabetic and antioxidant properties (Dhanukar, Kulkarni, Rege, 2000 ▶). These medicinal plants were used in the ancient Indian system of medicine such as ayurvedha and siddha for the treatment of madhu meha (diabetes mellitus) from time immemorial. In the present study one such reputed medicinal plant, *Tinospora cordifolia* (Family-Menispermaceae; Guduchi -Hindi), was selected for which previous records were reported to have most of the beneficial medicinal property (Singh et al, 2003 ▶) such as therapeutic supplement for pregnant diabetic rats (Shivananjappa and Muralidhara, 2012 ▶), a major work done with its alkaloidal fraction (Patel and Mishra, 2011 ▶) and aqueous portion of the stem (Sreenivasa Reddy et al, 2009 ▶), and with various extracts such as hexane, ethyl acetate, and methanol (Rajalakshmi et al., 2009 ▶). In the current study, the main aim is to elucidate the role of antioxidant potentials of the stem stalk of *T. cordifolia *via its antioxidant capacity and its potency, thereby hypoglycemic activity (Sangeetha et al, 2011 ▶) better than standard glibenclamide (Prince et al., 2004 ▶). Therefore, we made a cut sediment portion of aqueous soaked stem extract of *T. cordifolia* named as Guduchi satwa (sediment salt), composed of polysaccharide which consists chiefly of 1 4 linked glucan with occasionally branched points (Rao and Rao, 1981 ▶) and was used by ancient Indians for treating diabetes (Folklore medicine) for investigating its scientific evaluation of antioxidant activities *in vitro* (chemical reaction) and *in vivo* (experimental design) in diabetic rats.

## Materials and Methods

**Plant collection**

Dried stems of *Tinospora cordifolia* were collected from Irulars Tribal Women Welfare Society (ITWWS), Thandarai, Thirukazhukundram, a southern forest region of Tamil nadu, India. The pharmacognostical identification was done by Plant Anatomy Research Centre, Chennai. A specimen of the plant was kept in the Department of Pharmacology, C. L. Baid Metha college of Pharmacy, Chennai (Specimen No. CLBMCP/102/2005).

**Preparation of plant extract**

Around 2 kg of cut stems were grilled and grounded to a coarse powder and soaked in 1000 ml of distilled water and kept macerated for 24 hr. Next day, the top layer was decanted in a separate vessel (leaving the debris to filter off) and evaporated in a hot water bath at 100 ^o^C and reduced to 70 ^o^C following thick concentrate, to avoid escaping of thermolabile constituents. This portion is considered as water soluble portion which was admixed with sedimented portion in 1:3 ratio, after washing 2-3 times with fresh distilled water to avoid cell debris. The final sedimental extract of *Tinospora cordifolia* (SETc) was prepared as test drug for further screening. 

**Chemical and reagents**

All Chemicals and reagents were purchased from the local markets, Chennai; Sigma-Aldrich Laboratories, Mumbai; SRL Laboratories, Delhi, and S. D. Fine chemicals, Mumbai.

**Instruments**

Ascensia one touch glucometer and strips (Code no: 3110), were used for glucose estimation. All the analytical instruments and surgical equipments were of well-known manufacturers. 

**Animals**

Male Sprague dawley rats (200-250 g) were purchased from King’s Institute, Guindy, Chennai, and were experimented (CLBMCP/131/IAEC/41) under CPSCEA guidelines. All rats were randomly selected, segregated and acclimatized for a period of one week with 12 hr day light and 12 hr dark cycle, food and water ad libitum.

**Phytochemical analysis**

A portion of the test drug was subjected to preliminary phytochemical analysis using standard procedure (Harbone, 1974 ▶).

**Inhibitory concentration of** ***Tinospora cordifolia*** **-** ***in vitro*** **chemical analysis**

*DPPH radical scavenging activity* *(*Cotelle et al., 1996 ▶*) was examined as follow; *

To 0.2 ml of methanolic solution of DPPH (200 μM), 2.8 ml of test extracts (3 μg) ,dissolved in methanol, were added. Test extracts were prepared in different concentrations (3, 30, 60, 90, and 120 μg). The solutions were incubated at 37 °C for 30 min, absorbance measured at 517 nm. 

**Nitric oxide scavenging activity (**Sreejayan and Rao, 1997 ▶**)**

Nitric oxide radicals were generated from sodium nitroprusside solution at physiological pH. One ml of sodium nitroprusside (10 mM) was mixed with 1 ml of the test extracts/ascorbic acid (3 μg) in phosphate buffer (pH 7.4). The test extracts were prepared in different concentrations (3, 30, 60, 90, and 120 μg). The mixture was incubated at 25 ^o^**C** for 150 min. To 1.0 ml of the incubated solution, 1 ml of Griess’ reagent (1% sulphanilamide, 2% o-phosphoric acid, and 0.1% naphthyl ethylene diamine dihydrochloride) were added. Absorbance was read at 546 nm and percentage inhibition was calculated.

**Superoxide radical scavenging activity**

This activity was measured by the reduction of nitro blue tetrazolium (NBT) according to a previously reported method (Liu and Ng, 2000 ▶). The non-enzymatic phenazine methosulfate-nicotinamide adenine dinucleotide (PMS/NADH) system generates superoxide radicals, which reduce NBT to a purple formazan. 

4 ml reaction mixture contained phosphate buffer (20 mM, pH 7.4), 1 ml of NADH (73 μM), 1 ml of NBT (50 μM), 0.1 ml of PMS (15 μM), and 1.9 ml of various concentrations (3, 30, 60, 90, and 120 μg) of sample solution. After incubation for 5 min at ambient temperature, the absorbance at 560 nm was measured. All tests were performed six times.

**Hydroxyl radical scavenging activity** **(**Halliwell and Auroma, 1987 ▶**)**

One ml of the reaction mixture contained 100 μl of 28 mM 2-deoxy-2-ribose (dissolved in phosphate buffer, pH 7.4), 500 μl solution of various concentrations of Tinospora cordifolia (3, 30, 60, 90, and 120 μg), 100 μl of 200 μM FeCl_3_ and 100 μl of 1.04 mM EDTA, 100 μl H_2_O_ 2_ (1.0 mM), and 100 μl ascorbic acid (1.0 mM). After an incubation period of 1 hour at 37 °C the extent of deoxyribose degradation was measured by the TBA reaction. Absorbance was measured at about 532 nm against the blank solution. Vitamin E was used as a positive control. 

The percentage inhibition of all the assays, was calculated by comparing the results of the test with those of the control using the formula, percentage inhibition=absorbance of control - absorbance of test / absorance of control X 100.

**Reductive ability** **(**Jay Prakash et al., 2000 ▶**)**

Reducing power of the test extracts was determined based on the ability of antioxidants to form coloured complex with potassium ferricyanide, TCA and FeCl3. One ml of the test extracts (100-800 μg) /ascorbic acid (20 μg) in ethanol was mixed with 2.5 ml potassium ferricyanide (1%) and 2.5 ml of phosphate buffer (pH 6.6). The mixture was incubated at 50 ^o^C for 20 min. 2.5 ml TCA (10%) were added to it and centrifuged at 3000 rpm for 10 min. Two and a half ml of the supernatant was mixed with 2.5 ml water and 0.5 ml FeCl3 (0.1%). Absorbance was measured at 700 nm.

***In vivo*** **antioxidant study with SETc**

*Induction of experimental diabetes*

Sprague dawley rats (200-250 g) were fasted for 16 hours before the induction of diabetes with Streptozotocin (STZ). Animals were injected intraperitoneally with freshly prepared solution of STZ (45 mg/ml in 0.01 m citrate buffer, pH 4.5). The diabetic state was assessed in STZ-treated rats by measuring the non-fasting serum glucose concentration 48 hours post STZ injection. Only rats with serum glucose levels greater than 200 mg/dl were selected and used in this experiment (Soon and Tan, 2002).

***Treatment period***

A period of 30 days treatment with SETc of 1000 mg/kg/p.o., as dose fixed from the incremental dose finding procedure, studied earlier (Kannadhasan and Venkataraman, 2011 ▶) was carried out. On 31^st^ day, animals were sacrificed by anesthesia after 4 hours fasting, immediately followed by abdominal incision and removal of organs for *in vivo* antioxidant study and a portion of those were subjected to histopathological examination.

**Lipid peroxidation**

*Tissue Lipid peroxidation*

The TBARS levels measured as an index of malondialdehyde (MDA) production were determined (Uchiyma and Mihara, 1978 ▶). MDA, an end product of lipid peroxidation reacts with thiobarbituric acid to form a red coloured complex. The measurement of MDA levels by thiobarbituric acid reactivity is the most widely used method for assessing lipid peroxidation. Briefly, 1 g of the liver and kidney samples were homogenized in 4 ml of 1.15% ice cold KCl using a homogenizer to form a 25% (w/v) homogenate. To 0.1 ml of 25% homogenate, 0.2 ml of 8.1% dodecyl sodium sulphate salt (SDS), 1.5 ml of 1% phosphoric acid, 0.2 ml of distilled water, and 1.0 ml of 0.6% 2-thiobarbituric acid (TBA) were added. The mixture was heated in a boiling water bath for 45 minutes. Subsequently, the heated mixture was cooled in an ice bath, followed by an addition of 4.0 ml of n-butanol to extract the cold thiobarbituric acid reactants. The optical density of the n-butanol layer was determined at 353 nm after centrifugation at 2500 rpm for five minutes and expressed as nmol MDA/25 mg wet weight.

**Plasma lipid peroxidation (**Yagi, 1976 ▶**) **

To 0.2 ml of plasma, 4.0 ml of 3 N sulphuric acid was added, mixed well and 0.5 ml of 10% phosphotungstic acid was added. The contents were centrifuged and the supernatant was discarded. The sediment was mixed with 2.0 ml of N/12 H_2_SO_4 _and 0.3 ml of phosphotungstic acid. The mixture was centrifuged and sediment was dissolved in 4.0 ml of distilled water and to this, 1.0 ml TBA reagent was added and the contents were treated in a boiling water bath for 60 min. After cooling, 5.0 ml of n-butanol was added and the contents were shakened vigorously. Then it was centrifuged for 20 min and supernatant was read at 535 nm. Standards were also processed in similar manner.

Plasma lipid peroxides values are expressed as mg/dl.

***In vivo*** **antioxidant activity of SETc in normal and diabetic rats**

*Estimation of Enzymic antioxidants Superoxide dismutase (*Marklund and Marklund, 1974 ▶*)*

To 1.0 ml of the sample, 0.25 ml of absolute alcohol and 0.15 ml of chloroform was added. After 15 minutes of shaking in a mechanical shaker, the suspension was centrifuged and the supernatant obtained was constituted in the extract. The reaction mixture for auto oxidation consisted of 2 ml of buffer (Tris HCL 8.2), 0.5 ml of 2 mM pyrogallol, and 1.5 ml of water. Initially, the rate of auto oxidation of pyrogallol was noted at an interval of 1 min for 3 min.

The assay mixture for the enzyme contained 2 ml of 0.1 M Tris HCL buffer; 0.5 ml of pyrogallol, aliquots of the enzyme prepared and water to give a final volume of 4 ml. The rate of inhibition of pyrogallol auto oxidation after the addition of enzyme was noted. The enzyme activity is expressed in terms of units/min/mg protein in which one unit corresponds to the account of enzyme required to bring about 50% inhibition of pyrogallol auto oxidation.

***Catalase (***Sinha, 1972 ▶***)***

One-tenths of a ml of the homogenate was taken to which 1 ml of phosphate buffer and 0.5 ml of H_2_O_2_ was added. The reaction was arrested by the addition of 2.0 ml dichromate-acetic acid reagent. Standard H_2_O_2_ in the range of 10 to 160 µmoles and 4 to 10 µmoles were taken and treated similarly. The tubes were heated in a boiling water bath for 10 min. the green colour developed was read at 570 nm.

Catalase activity in tissue homogenate is expressed as nmoles of H_2_O_2_ consumed/min/mg protein at 37 ^o^C.

**Estimation of non-enzymic antioxidants vitamin C (ascorbic acid)**

Vitamin C was estimated followed by the method of Omaye et al., 1979 ▶. Aliquots of homogenate were precipitated with 5% ice cold tricarboxylic acid and centrifuged for 20 min at 6500 rpm. One-tenths of a ml of the supernatant was mixed with 0.2 ml of DTC (2,4 dinitrophenyl hydrazine: thiourea: copper sulphate) and incubated for 3 hr at 37^ o^C. Then 1.5 ml of ice cold 65% H_2_SO_4_ was added, mixed well and the solution was allowed to stand at room temperature for additional 30 min. Absorbance was determined at 520 nm.

Ascorbic acid values are expressed as µg/mg protein.

**Vitamin E (α-Tocopherol)**

Vitamin E was estimated by the method of Desai et al., 1984 ▶.

**Saponification and extraction**

To 500 mg of the tissue, 5.0 ml of isotonic KCl was added and homogenized. To 1.5 ml of homogenate 1.0 ml of ethanol and 0.5 ml of 25% abscorbate were added and pre-incubated at 70 ^o^C for 5 min in glass-stoppered tubes. To this, 1.0 ml of saturated KOH was added and mixed again. This mixture was further incubated at 70 ^o^C for 30 min. The tubes were immediately cooled in an ice water bath and 1.0 ml of distilled water and 4.0 ml of purified hexane were added. The tubes were shaken vigorously for 2 min and centrifuged at 1500 rpm for 10 min to separate the phases.

**Estimation**

Three ml aliquots of hexane extract was pipetted out into suitable reaction tubes and evaporated to dryness under nitrogen. The residue was then carefully dissolved in 1.0 ml of purified ethanol. The tubes containing α-Tocopherol standard were treated in the same way as test samples. To all the tubes, including a reagent blank, 0.2 ml of 0.2% bathophenanthroline reagent was added and the contents of the tubes were thoroughly mixed. The assay proceeded very rapidly from this point and care was taken to reduce unnecessary exposure to direct sunlight. 

Two-tenths of a ml of ferric chloride reagent was added and the tubes were mixed by vortexing. After 1 min, 0.2 ml of orthophosphoric acid was added and the tubes were thoroughly mixed again. 

The absorbance was read at 536 nm.Vitamin E value is expressed as mg/gm tissue.

**Histopathological studies (**Kanai Mukherjee, 1989 ▶**)**

The dissected samples of pancreas, liver and kidney from each group of animals were collected in 10% formalin solution and stained with hemotoxylin and eosin for preparation of section by using of microtome in Vaishnave Clinic, Chennai – 17.

## Results

**Preparation of plant extract**

535 g of sedimental extract and 15 g of water soluble portion dried under vacuum at 37 ^o^C and obtained a total quantity of 550 g (SETc), which were used for the whole study.

**Preliminary phytochemical screening**

From the phytochemical analysis, it was observed that the sedimental extract of *Tinospora cordifolia* (SETc) showed the presence of active chemical ingredients as reported previously for various parts of the plant (Table 1).

**Antioxidant property of SETc by using** ***in vitro*** **– chemical methods**

**Figure 1:**
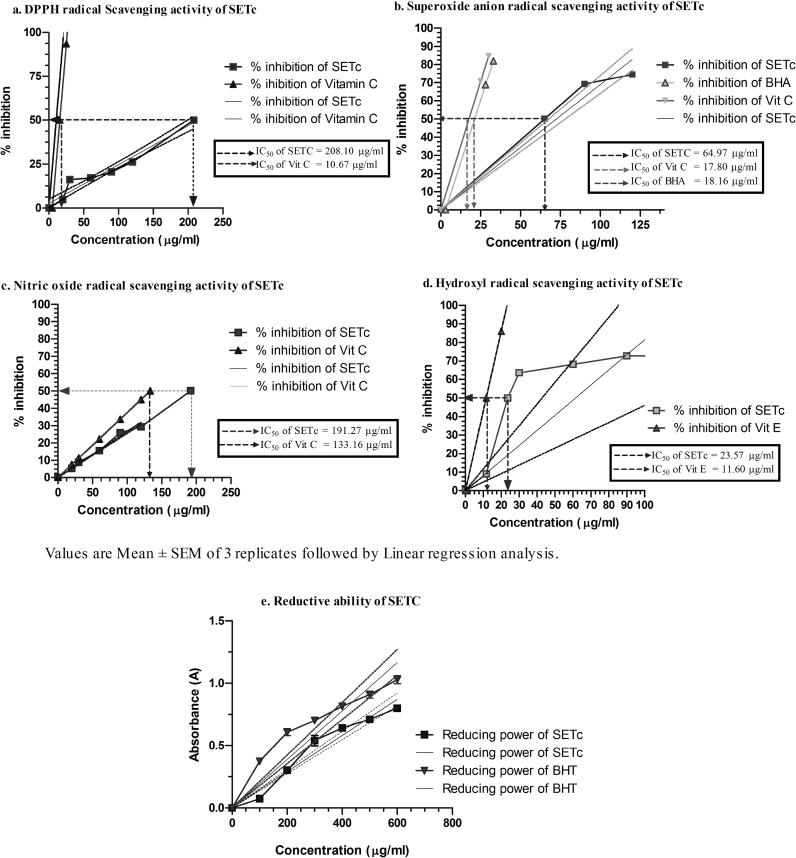
Antioxidant capacity – *in vitro* chemical analysis with SETc. Values are mean±SEM of 3 replicates followed by liner regression analysis. Increase in reducing power is directly proportional to increase in absorbance of the sample used at 700 nm

SETc in different concentrations were tested for their antioxidant activity in five different *in vitro* models (Figure 1).

**Table 1:** Phytochemical screening of sedimental extract of *Tinospora cordifolia*

**Active Ingredients**	**Inference**
**Alkaloids**	+++
**Carbohydrates & Glycosides**	+
**Specific Glycosides**	-
**Cardiac Glycoside**	+
**Reducing sugars**	-
**Steroids**	+
**Saponins**	+
**Tannins**	++
**Condensed tannins**	+
**Pseudo tannins**	-
**Flavonoids**	++

**DPPH radical scavenging activity of SETc**

The maximum percentage inhibition of DPPH by SETc was 26.57±0.254 at 120 µg concentration, whereas standard ascorbic acid showed 93.64±0.239 percentage inhibition of the DPPH at 20 µg (Figure 1.a). The IC_50_ value of SETc was found to be 208.10 µg as obtained from the linearity curve. This is the concentration at which the free radical DPPH is scavenged.

**Superoxide radical scavenging activity of SETc**

The percentage inhibition of superoxide radical generation at 120 µg was found to be 74.49±0.286. However, Ascorbic acid and BHA showed percentage inhibition of 70.66±0.254 and 68.86±0.071, respectively at 25 µg each which is similar to the inhibition that produced by SETc at 120 µg and 90 µg, respectively. The IC_50_ value of SETc was found to be 65.87 µg against 17.80 and 18.16 µg for standard vitamin C and BHA, respectively (Figure 1.b).

**Nitric oxide scavenging activity of SETc**

In the nitric oxide model, the maximum percentage inhibition of SETc on nitric oxide radicals was 29.25±0.245 at 120 µg concentration. However, ascorbic acid at 20 µg caused only 7.51±0.015 percentage inhibition which was achievable by a concentration of 30 µg of SETc. The IC_50_ value of SETc from the linearity curve was found to be 191.27 µg whereas vitamin C showed IC_50_ at 133.16 µg (Figure 1.c).

**Hydroxyl radical scavenging activity of SETc**

The hydroxyl radical scavenging activity of SETc at 120 µg was found to be 73.62±0.456 percentile, a very near value to that of the standard vitamin E at 20 µg (86.03±0.619). The IC_50_ values of SETc and vitamin E were found to be 23.57 and 11.60 µg, respectively (Figure 1.d).

**Reductive ability**

The reducing ability of SETc was studied using the gradient concentrations of SETc ranging from 100 to 600 µg/ml. The maximum concentration of SETc (i.e., 600 µg/ml) showed a maximum absorbance of 0.799±1.578 the value which was closer to 400 µg of BHT (Figure 1.e).

***In vivo*** **Antioxidant property of SETc**

*Lipid peroxidation*

**Figure 2:**
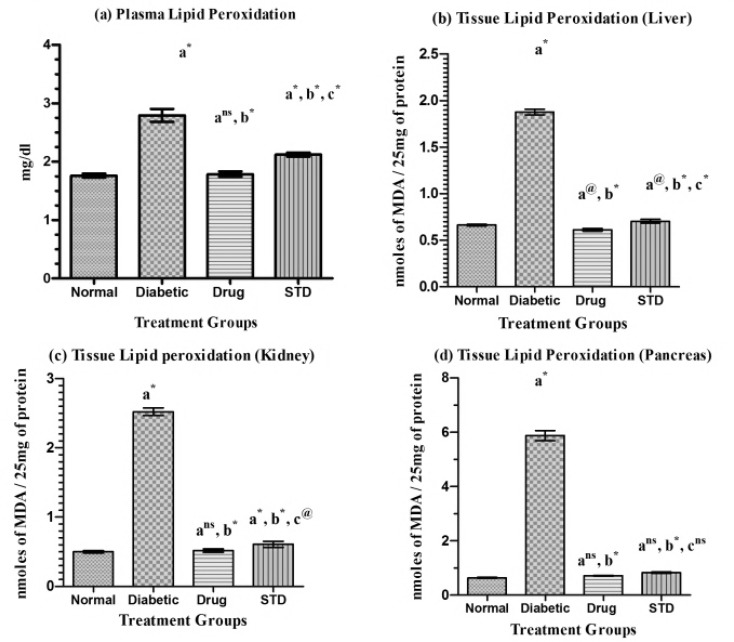
Lipid peroxide activity of normal, diabetic, and diabetic rats treated with SETC after 30 days.

From the Figure 2, it was observed that the plasma lipid peroxidation and tissue lipid peroxidation level of liver, kidney, and pancreas of SETc (1000 mg/kg/p.o,) treated group were found to be reduced as compared with that of the diabetic control (p<0.001) and also found to have moderate difference (p<0.01) in liver and no significant difference (p=ns) in kidney and pancreas peroxide level as compared with that of the normal and standard drug treated groups.

n = 6; p<0.05 is considered as statistically significant. Values are expressed as mean±S.E.M. using one-way ANOVA followed by Tukey’s multiple comparison method. Units are expressed as: Plasma Lipid Peroxides: mg/dl; Tissue Lipid Peroxides: nmoles of MDA liberated/min/mg protein.

a = normal control Vs diabetic control, test drug and standard. b = diabetic control Vs test drug and standard. c = test drug Vs standard. * = p<0.001; @ = p<0.01; # = p<0.05; ns = non significant.

**Enzymic and non-enzymic antioxidants**

**Figure 3:**
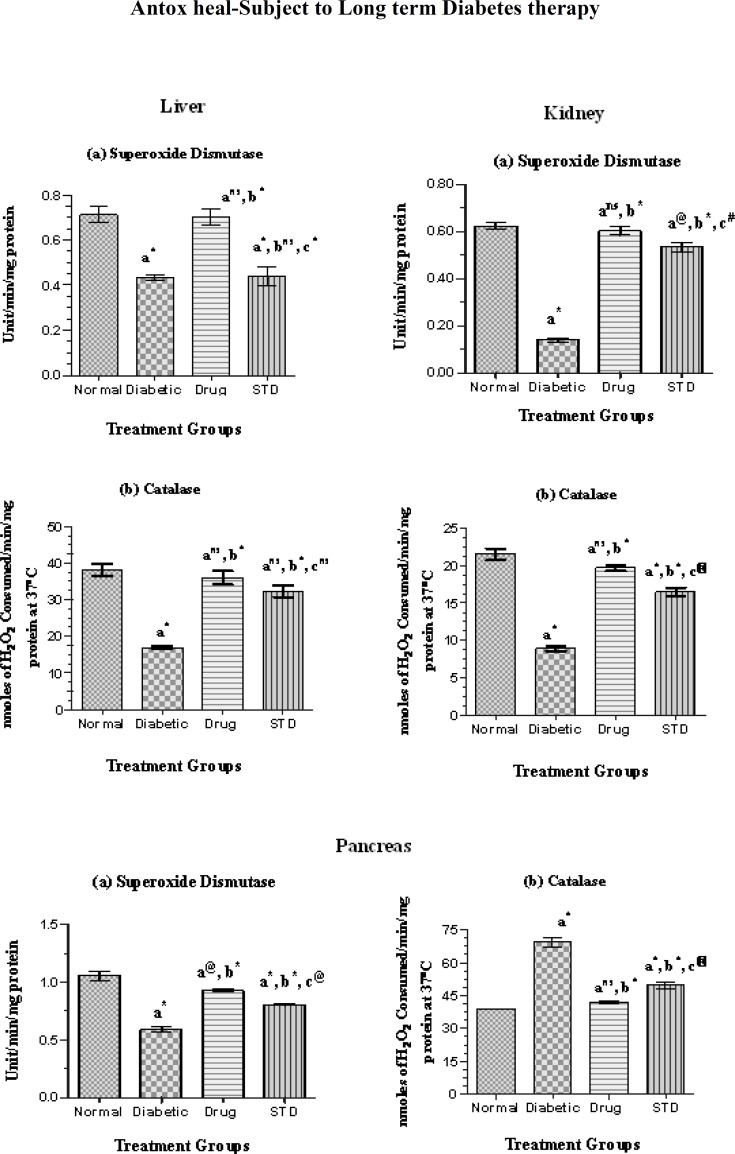
Enzymic antioxidant levels in normal, diabetic, and diabetic rats treated with SETc after 30 days treatment.

**Figure 4:**
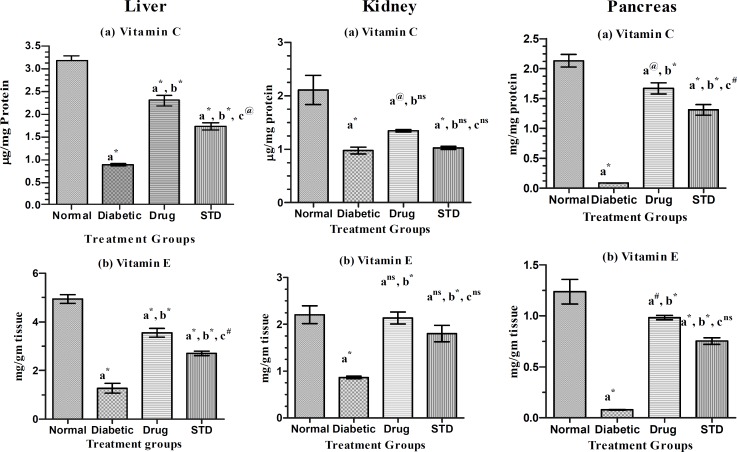
Non-enzymic antioxidant levels in normal, diabetic, and diabetic rats treated with SETc after 30 days treatment. n=6; Values are expressed as mean ± S.E.M. using one-way ANOVA followed by Tukey’s multiple comparison method.

The enzymic and non-enzymic antioxidant level in the liver of diabetic rats treated with SETc was shown in Figures 3 and 4.

**Liver**

The SOD level of SETc treated rats was found to possess significant difference (p<0.001) as compared with that of normal control and possessed no significant difference (p=ns) as compared with that of diabetic control and standard drug treated groups (Figure 3a).

The CAT level of SETc treated rats was found to show most significant increase (p<0.001) as compared with that of the diabetic control and possessed no significant difference (p=ns) to that of the standard group (Figure 3b). There was significant difference in vitamin C and vitamin E levels of SETc treated group as compared with that of the normal and diabetic control (p<0.001) and there was a moderate and less significant increase in the level of vitamin C and vitamin E levels (p<0.01) and (p<0.05), respectively as compared with that of the standard drug treated group (Figures 4a and b).

**Kidney**

The SOD level of SETc treated group was found to have significant increase (p<0.001) as compared with that of the normal, diabetic control, and standard drug treated groups (Figure 3a). The catalase enzymic level of SETc treated group was found to have significant increase (p<0.01) as compared with that of the diabetic control and significant difference (p<0.01) to that of the standard drug treated group (Figure 3b). As shown in Figure 4a, the vitamin C level of SETc treated group was found to have no significant difference as compared with that of the diabetic control and standard drug treated group (p=ns). 

The vitamin E level of SETc treated group was found to possess no significant difference as compared with that of the normal control and standard drug treated groups and there was most significant increase (p<0.001) as compared with that of the diabetic control (Figure 4b). 

**Pancreas**

The SOD level of SETc treated rats was found to have significant increase (p<0.001) as compared with that of the diabetic control (Figure 3a). There was significant difference as compared with that of the standard drug treated rats (p<0.001). There was a significant reduction in the enzymic catalase level of SETc treated group (Figure 3b), as compared with that of the diabetic control (p<0.001) and values reduced near to normal control (p=ns). From Figure 4a, there was a significant increase in the vitamin C level of SETc treated diabetic rats was observed as compared with that of diabetic control (p<0.001) and standard drug treated rats (p<0.05). Vitamin E level of SETc treated diabetic rats showed a significant increase (p<0.01) as compared with that of diabetic control and no significant difference (p=ns) to that of the standard drug treated rats (Figure 4b). 

n = 6; p<0.05 is considered as statistically significant. Values are expressed as mean±S.E.M. using one-way ANOVA followed by Tukey’s multiple comparison method. Units are expressed as: Superoxide Dismutase (SOD): Unit/min/mg protein; Catalase (CAT): nmoles of H_2_O_2_ consumed/min/mg protein at 37^o^ C.

a = normal control Vs diabetic control, test drug and standard; b=diabetic control Vs test drug and standard; c = test drug Vs standard. *= p<0.001; @ = p<0.01; # = p<0.05; ns=non significant.

Figure 4. Non-enzymic antioxidant levels in normal, diabetic, and diabetic rats treated with SETc after 30 days treatment. n=6; Values are expressed as mean ± S.E.M. using one-way ANOVA followed by Tukey’s multiple comparison method.

Units are expressed as: Vitamin C (Ascorbic acid): µg/mg protein; Vitamin E (α- Tocopherol): mg/gm tissue.

a = normal control Vs diabetic control, test drug and standard; b = diabetic control Vs test drug and standard; c = test drug Vs standard. *= p<0.001; @ = p<0.01; # = p<0.05; ns = non significant.

**Histopathology study after 30 days treatment with SETc in diabetic rats**

**Figure 5:**
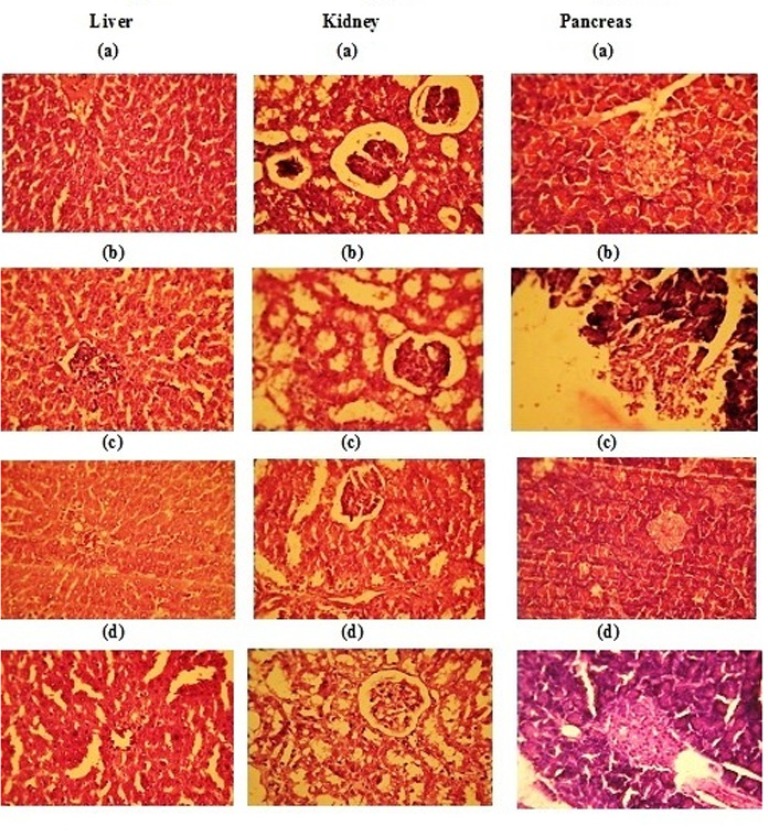
Histopathology of major organs after days treatment with SETc. (a): Normal control; (b): Diabetic control; (c): SETc(1000mg/kg/ p.o.); (d): Standard glibenclamide (600 µg/kg/ p.o.)

The micro anatomical changes in the liver, kidney, and pancreas of diabetic rats treated with SETc for 30 days are depicted in Figure 5.

**Liver**

A strong divergence was observed in diabetic rats’ hepatocytes from the normal ones. The untreated diabetic rats showed congestion of veins surrounded by hepatocytes and necrosis with inflammation. The liver section of SETc treated rats showed less inflamed area along with normal hepatocytes that might be the repairing sign of damaged cells caused by STZ induction as compared with that of the standard drug treated group.

**Kidney**

The kidney of the diabetic control showed congestion in the glomeruli with tubular epithelial damage. And there were almost-normal glomeruli with regenerating tubular epithelium that were observed in SETc treated group, more comparable with normal control than standard drug treated group. 

**Pancreas**

The pancreas of the diabetic control showed fatty infiltration of islet cells whereas SETc treated group showed less hyperplastic islet cells as compared with that of standard drug treated group.

Units are expressed as: Vitamin C (Ascorbic acid): μg/mg protein; Vitamin E (α- Tocopherol): mg/gm tissue.

a = normal control Vs diabetic control, test drug and standard; b = diabetic control Vs test drug and standard; c = test

drug Vs standard. *= p<0.001; @ = p<0.01; # = p<0.05; ns = non significant.

## Discussion

**Antioxidant property of SETc by using** ***in vitro*** **– chemical methods**

The test compound SETc exhibited *in vitro* antioxidant activity in a concentration dependant manner up to the given concentrations studied. DPPH is a relatively stable nitrogen centered free radical that easily accepts an electron or hydrogen radical to become a stable diamagnetic molecule. DPPH radicals react with suitable reducing agents as result of which the electrons become paired off forming the corresponding hydrazine. The solution therefore loses colour stochiometrically depending on the number of electrons taken up (Blois, 2001 ▶). The IC_50_ value of SETc was found to be 208.10 µg as obtained from the linearity curve. This is the concentration at which the free radical DPPH is scavenged.

 Superoxide anion radicals are produced endogenously by flavoenzymes such as xanthine oxidase, which converts hypoxanthine and subsequently to uric acid in ischemia-reperfusion. Superoxide is generated in vivo by several oxidative enzymes, including xanthine oxidase. In the PMS-NADH-NBT system, superoxide anion derived from dissolved oxygen by PMS-NADH coupling reaction reduces NBT (Arulmozhi et al., 2007 ▶). The decreased absorbance at 560 nm indicates the consumption of superoxide anions in the reaction mixture by SETc. 

 Nitric oxide is an important chemical mediator generated by endothelial cells, mesophages, and neurons and involved in the regulation of various physiological processes. Excessive concentration of nitric oxide produces serious cytotoxic effects observed in various disorders including AIDS, cancer, alzheimer’s, and arthritis (Sainanai et al., 1997 ▶). Oxygen reacts with NO^-^ to generate nitrite and peroxy nitrite anions, which act as free radicals. In this study, the nitrite produced by incubation of solutions of sodium nitroprusside in standard phosphate buffer at 25 ^o^C was reduced by SETc, which might be due to antioxidant principles of the extract, competing with oxygen to react with nitric oxide thereby inhibiting the generation of nitrite. Hydroxyl radicals are the major reactive oxygen species causing lipid peroxidation and enormous biological damage (Aurand et al., 1977 ▶).

The *in vitro* antioxidant assay using SETc showed IC_50 _values more or less equal to standard drug vitamin C in nitric oxide scavenging activity whereas IC_50_ values were very high in comparison to standard drugs in *in vitro* DPPH and superoxide radicals scavenging activity. The reductive ability of SETc was comparable to that of BHT, the standard chemical used for comparison. The overall *in vitro* antioxidant capacity of 1000 mg/kg/day of SETc was found to be 2046 times of its own therapeutic advantage as an antioxidant which was further to be proceeded for its ameliorating effect over diabetes in future.

***In vivo*** **antioxidant property of SETc**

Lipid peroxidation is a free radical mediated process leading to oxidative deterioration of polyunsaturated lipids. Under normal physiological conditions, low concentrations of lipid peroxide are found in plasma and tissues. The possible source of oxidative stress in diabetes includes shifts in redox balance resulting from altered carbohydrate and lipid metabolism, increased generation of reactive oxygen species, and decreased level of antioxidant defenses such as GSH and ascorbic acid (Baynes, 1991 ▶).

The increase in lipid peroxide level due to streptozotocin was not much in the liver and kidney, probably because the catalase activity is high enough to counteract the oxygen stress. On the other hand, the level of lipid peroxides in the pancreas was significantly elevated by streptozotocin, probably because of a marked reduction of catalase. Because the activities of antioxidant enzymes in the pancreas are relatively lower than those in the other organs (Wohaieb and Godin, 1987 ▶), radicals derived from streptozotocin or streptozotocin-induced diabetes may selectively attack the pancreas which leads to oxidative stress, thereby evoking the defense mechanism. In nature, the catalase activity is high in the liver, medium in the heart, low in the pancreas (Wohaieb and Godin, 1987 ▶), and high in the kidney (Suryanarayana et al., 2007 ▶).

Such marked alterations in the plasma and tissue lipid peroxides level of liver, kidney, and pancreas in STZ-induced diabetic rats were effectively replaced with normal value ranges after 30 days of treatment with SETc (1000 mg/kg/p.o.). From the results, it was very clear that the test drug SETc was found to show its potential anti-oxidative role which compensates the enzymes level elevated as a defense mechanism over hyperglycemia- induced systemic and tissues specific oxidative stress, which was supported from the previous report for the effect of *T. cordifolia* for its possible restoration of antioxidant defence against alloxan-induced tissue damages (Stanley Mainzen Prince et al., 2004 ▶). 

Oxidative stress in diabetes co-exists with altered antioxidant systems, both enzymatic and non-enzymatic. However, the connection between altered antioxidant enzymes and increased oxidative stress is not straightforward, as changes (increase or decrease) in the activities of antioxidant enzymes are not always unidirectional. Thus while in some studies the activities of SOD, CAT, GPx, and GST in diabetes mellitus showed reductions in the levels of these enzymes (Okutan et al., 2005 ▶; Ozkaya et al., 2002 ▶; Sugiura et al., 2006 ▶), some other studies reported increases in the activities of these enzymes in STZ-induced diabetes (Okutan et al., 2005 ▶; Yilmaz et al., 2004 ▶). Furthermore, changes in antioxidant enzymes may be organ specific. This may be due to an organ-specific response to hyperglycemia-induced oxidative stress. Nevertheless, the relative change in activity in diabetic tissues compared with control tissues might indicate an altered antioxidant system. 

SOD catalyses the conversion of super oxide anion to hydrogen peroxide and oxygen. In two studies (Matkovica, 1977 ▶; 1982), researchers found that rats with STZ-induced diabetes had decreased SOD activity in liver, kidney, spleen, heart, pancreas, skeletal muscle, testis, and erythrocytes. Catalase is a haem-containing ubiquiter enzyme and in eukaryotes, it is found in peroxisomes. This enzyme probably serves to degrade hydrogen peroxide produced by peroxisomal oxidases to water and oxygen. The enzymic level of catalase was found to be increased in diabetic rats, especially in heart and pancreas which shows the oxidant–antioxidant imbalance (Erika et al., 1999 ▶). Changes in the levels of non-enzymatic antioxidants have been observed in diabetic condition. Vitamin C is a water soluble antioxidant that primarily scavenges oxygen free radicals. Vitamin C has been reported to contribute up to 24% of the total peroxyl radical–trapping antioxidant activity (Atanaisu et al, 1998 ▶). Decreased level of vitamin C observed in diabetic condition might be due to increased utilization of vitamin C in deactivation of the increased levels of the reactive oxygen species (Chattejee and Nandi, 1991 ▶).

Vitamin E is also an important radical scavenging antioxidant that interrupts the chain reaction of lipid peroxidation by reacting with the lipid peroxyl radicals (Takenaka et al., 1991 ▶). Increased utilization of the vitamin E in the plasma was due to the increased production of lipid peroxides, as a result of decreased non enzymic level of vitamin E in the tissues (Senthil kumar and Subramaniam, 2007 ▶). It was observed from the present study that SOD, CAT, vitamin C and vitamin E levels of tissues including; liver, kidney, pancreas, and heart of diabetic rats were normalized after SETc treatment for a period of 30 days. In this context, feeding SETc appears to have resulted in considerable reversal, but not complete normalization, of antioxidant enzymes that were altered in diabetic tissues.

**Histopathological study**

The SETc treatment with all its antioxidant capacity was evidenced from its positive impregnation over destructed cells by streptozotocin directly and by metabolic imbalance/modulation of gluconeogenic enzymes (Puranik et al, 2009). The resulting changes observed for the period of 30 days of drug treatment in respective animals will be a resurrecting tool for long term complications in diabetes.

The present study on the antioxidant activity on both *in vitro* and *in vivo* of the SETc in diabetic rats concludes: SETc treatment showed significant free radical scavenging activity *in vitro* antioxidant assay. SETc treatment increased the levels of both enzymic and non-enzymic antioxidant levels in tissues. SETc treatment protected the tissues from lipid peroxidation in diabetic rats. The histopathology study clearly establishes the nontoxic and protective effect of SETc in the internal organs such as liver, kidney, and pancreas. The overall studies clearly show the antioxidant capacity of SETc around 2046 times in crude form whose particular ingredients responsible for such property and potency have to be established. Furthermore, impregnation of SETc at dose 1000 mg/kg/p.o., on diabetes therapy is yet to be determined.
